# Social activity promotes resilience against loneliness in depressed individuals: a study over 14-days of physical isolation during the COVID-19 pandemic in Australia

**DOI:** 10.1038/s41598-022-11315-4

**Published:** 2022-05-03

**Authors:** Julie L. Ji, Julian Basanovic, Colin MacLeod

**Affiliations:** School of Psychological Science, University of Western Australia, M034 35 Stirling Highway, Perth, WA 6009 Australia

**Keywords:** Public health, Human behaviour

## Abstract

Loneliness is a subjectively perceived state of social isolation that is associated with negative emotional, cognitive, and physical health outcomes. Physical distancing and shelter-in-place public health responses designed to curb COVID-19 transmission has led to concerns over elevated risk of loneliness. Given that physical isolation does not necessitate social isolation in the age of digital communication, this study investigated the relationship between the frequency of social interaction and loneliness over a two-week period in people engaging in physical distancing and examined whether this relationship was moderated by physical isolation level, age, or depression. A self-selected sample of *N* = 469 individuals across Australia who were engaged in physically distanced living completed daily surveys for 14-days during April to June of 2020. Multilevel modelling showed that more frequent social interaction with close, but not intermediate or distant contacts, was uniquely associated with lower loneliness. In addition, being younger, more depressed, more anxious, or having a mental health condition diagnosis (past or present) were also independently associated with higher loneliness. Critically, depression was the only significant moderator of the relationship between social interaction and loneliness over time, where more frequent social interaction with close contacts buffered against loneliness over time in high depression individuals only. The findings suggest that encouraging social activity with close contacts may promote resilience against loneliness in individuals with elevated depression symptoms.

## Introduction

Public health responses to the COVID-19 pandemic, such as “stay-at-home” orders, have disrupted the social lives of individuals worldwide on an unprecedented scale. As individuals and entire communities endure extended periods of physically-distanced living, there are serious concerns that physical isolation may result in adverse mental health outcomes, including elevated stress^1,2^, depression and anxiety^3,4^, and loneliness^5,6^.

Humans, like other social animals, have a fundamental need to feel a sense of belonging and connection to others^7^. The subjective perception of having unmet interpersonal needs, when one experiences a state of thwarted belonging, gives rise to the emotionally aversive state of “loneliness”^8^. While loneliness is theorized to be adaptive when signalling deficient social resources, as it can trigger responses to reinstate social connections, chronic loneliness is associated with harmful mental and physical health consequences^9^ and is theorised to be a key predictor of suicidality^10,11^.

Studies on loneliness during the pandemic has indicated that physically-distanced living due to lockdown restrictions is associated with elevated levels of loneliness relative to pre-pandemic levels in the US^5^, UK^12,13^, and Australia^14^. Between April and May of 2020, loneliness was the most widely reported personal stressor due to COVID-19 in Australia, with 25% of 19,385,000 respondents reporting suffering from loneliness^15^.

While lockdown living during the COVID-19 pandemic severely disrupts opportunities for face-to-face social interaction, this need not result in social isolation in the age of digital communication. Yet, despite concerns about the possibility of rising loneliness due to physical isolation, research has yet to investigate the manner in which social interaction frequency impacts experiences of loneliness across time during lockdown living, nor how this impact may differ amongst populations that vary in vulnerability. We will describe existing evidence for the association between social interaction and loneliness, and identify factors that may plausible moderate this association, before describing the specific hypotheses and predictions tested in the present study and the methodological design used to determine their validity.

### Social interaction and loneliness

Previous research has shown that social isolation independently predicts loneliness, over and above demographic and background factors^16^. Interacting and communicating with others is essential for building and maintaining social relationships and social connectedness. Although COVID-19-related lockdown restrictions can severely reduce opportunities for daily face-to-face interaction, continued social interaction is possible even during lock-down periods due to the ready availability of communication platforms that offer the opportunity to interact with others via video, voice, and text mediums. However, it is as yet unknown whether the *frequency* of socially interaction, and *with whom* the interaction occurs, serves to predict loneliness during periods of social-distancing behaviours engaged in response to the COVID-19 pandemic.

Thus, it is plausible that maintaining social interaction during physically distanced-living with close contacts specifically (immediate family, close friends, partner) may be more beneficial in buffering against loneliness over time, as compared to social interaction with other contacts. It is important to note that the amount of social interaction one engages in is not necessarily associated with level of loneliness^17^. Several studies have failed to find significant differences between lonely and non-lonely people in the amount of social activity engaged in, or the amount of time spent with other people^17,18^. However, a study by Jones^19^ showed that while the total amount of social interaction may not vary between lonely and non-lonely people, the social distance of the interaction contacts does. Specifically, non-lonely individuals had more interactions with close family and friends than lonely individuals, whereas lonely individuals had more interactions with strangers and acquaintances^19^.

In addition to the closeness of relationships with social interaction contacts, existing evidence indicates that the level of physical isolation experienced, age, and level of co-existing depression symptoms may plausibly impact the degree to which social interaction activities impact loneliness over time. These candidate moderating factors will now be described in turn.

### Social activity and loneliness—potential moderating factors

#### Physical isolation

One factor that may moderate the impact of social interaction on loneliness during the COVID-19 pandemic is the degree of physical isolation experienced by individuals. Evidence indicates that being alone does not necessarily result in feelings of loneliness^20^, and that levels of physical isolation and loneliness are often not correlated^21–23^. For example, individuals living in single-person households have reported higher levels of loneliness than those who live in multi-person households^24^, and a large (*N* = 15,530) nationally representative study from the UK identified living with a partner as a protective factor against loneliness during COVID-19^12^. It is therefore plausible that physical isolation from others, such as due to living alone and/or not going outdoors, serves as a moderating factor that increases the degree to which reduced social interaction serves to elevate loneliness across time during the COVID-19 period. This moderating impact could operate such that greater levels of physical isolation from others may serve to enhance the detrimental impact of low social interaction, or to diminish the beneficial impact of heightened social interaction, upon loneliness. If found to be the case, this would indicate that engaging in social interaction during physically-distanced living may be particularly important for those who are more physically isolated from others.

#### Age

Another factor that may moderate the impact of social interaction on loneliness during physical distancing is age. Research has shown that certain age groups are more prone to loneliness than others. For example, researchers have demonstrated that young people tend to report the highest levels of loneliness out of all age groups^25–27^. In the context of COVID-19, nationally representative data from the UK collected in the first half of 2020 indicates that young people are more likely to experience loneliness than any other age group^12^, consistent with other studies from the same time period^28^. However, older adults have also been known to experience elevated loneliness, as older adults are more likely to live in relative physical isolation^24^.

While both younger and older populations may be more vulnerable to loneliness than people mid-range in age, young people’s social interactions may be impacted more heavily by COVID-19 related changes. School and campus closures as well as loss of casual employment opportunities have severely reduced young people’s opportunities to develop and maintain social relationships, whereas these changes may have less impact on older adults’ social relationships. Moreover, social relationships with peers are of particular importance to young people^29^, outweighing familial relationships in terms of subjective importance^30^. Thus, reduced engagement in social interaction during the COVID-19 pandemic may be particularly impactful on younger people’s levels of loneliness.

#### Depression

Loneliness and depression are highly comorbid, sharing many affective, cognitive, and behavioural features^31^, and so depression is strongly linked to increased risk of loneliness. For example, US data from April 2020 revealed that 54.7% of lonely participants reported clinically significant levels of depression, compared to 15.3% in non-lonely participants^5^. Further, evidence shows that loneliness and depression exacerbate one another over time^32^ and addressing loneliness can help alleviate depression over time^33^.

Importantly, level of depression symptoms may moderate the relationship between social interaction and loneliness. Researchers have observed that individuals with elevated depression symptoms have stronger negative responses to experiences of social exclusion and rejection than do those with low depression symptoms, and also show stronger positive responses to experiences of positive social interaction, including a sense of belonging^34^. Thus, elevated depression symptoms may sensitize people to everyday experiences of both social isolation and social connection, suggesting that the detrimental impact of reduced social activity on loneliness during physically distanced-living may be greater for those with elevated depression symptoms than for those with low depression symptoms.

### The present study

Researchers have yet to determine the impact of social interaction with others, who vary levels of social closeness, upon loneliness during periods of physical-distancing during the COVID-19 pandemic. The nature of this relationship, and identification of the factors that moderate it, will be critical to investigators and end-users who wish to understand the determinants, and protective factors, of elevated loneliness.

For this reason, the present study had two aims. The study’s first aim was to investigate the relationship between social interaction frequency with close, intermediate, and distant social contacts and loneliness over time during COVID-19 pandemic-related lockdown living, over and above demographic, contextual, and general mental health variables. The study’s second aim was to determine whether the relationship between loneliness and social interaction with close, intermediate, and distant contacts across time is moderated by physical isolation, age, and depression. To achieve this, the study measured loneliness across a 14-day period of physically distanced-living during April to June 2020 amongst community-dwelling individuals across Australia and investigated the relationship between social interaction frequency and loneliness, and whether this relationship was moderated by physical isolation, age, or depression level, after controlling for the effects of gender, mental health diagnosis status, and duration of lockdown living.

#### Hypotheses

It was hypothesised that more frequent social interaction, particularly with close contacts, would be associated with lower levels of loneliness (Hypothesis 1). In addition, it was hypothesised that any demonstrated relationship between social interaction frequency and loneliness would be moderated by physical isolation, age, and depression. Specifically, it was predicted that the beneficial impact of more frequent social interaction on loneliness would be more pronounced for individuals who are more physically isolated, compared to those who are less physically isolated (Hypothesis 2); for individuals who are younger compared to those who are older in age (Hypothesis 3); and for individuals who experience relatively higher, as compared to lower, levels of depression symptoms (Hypothesis 4).

## Methods

### Study design

This study utilised a longitudinal design comprising daily surveys over a 14-day period with a self-selected sample from across Australia.

### Recruitment

Participants were made aware of the study via social media posts and announcements via radio and print news media that directed them to a study specific recruitment website. Study recruitment information specified the study’s inclusion criteria, which were (a) residing in Australia for the duration of the study; (b) engaged in physically distanced living at the time of the study, described as staying indoors (at home, hotel room, or other accommodation) and only going outside when necessary, such as for food shopping, exercise, or medical appointments, or going to work if working from home is not possible; and (c) aged 18+. Data was collected between 15th of April to 22nd July 2020 (5% April, 94% May, 0.6% June, and 0.4% July). In an attempt to flatten the curve following Australian’s first wave of COVID-19 infections during March 2020, all Australian states enacted strict stay-at-home orders throughout April and for most of May.

### Participants

All individuals provided electronic informed consent before completing any part of the study procedure. A total of *N* = 2470 individuals responded in part or whole to the study’s initial registration survey. Respondents were excluded from analysis if (a) they did not complete the registration survey; and (b) if they did not complete more than one weekly loneliness assessment across the duration of the study, resulting in *N* = 469 respondents included in data analysis for this study. The study protocol was approved by the University of Western Australia Human Research Ethics Committee (RA/4/20/6226). Informed consent was obtained from all participants. All study procedures adhered to the guidelines and regulations outlined in the Australian National Statement on Ethical Conduct in Human Research 2007, and in accordance with the Declaration of Helsinki.

### Materials

#### Loneliness

Loneliness was assessed weekly using the Thwarted Belongingness Scale (TBS; Ma et al., 2019), an 8-item scale that asks participants to rate their agreement with statements about perceived loneliness and unmet interpersonal needs (e.g. ‘I feel isolated’; ‘Nobody cares about me’). Each item was answered on a 7-point scale ranging from 1 (not at all true for me) to 7 (true for me) with total scores ranging from 8 to 56. Higher scores indicate greater thwarted belongingness. The TBS has demonstrated convergent validity and predictive validity with the Thwarted Belonging subscale of the Interpersonal Needs Questionnaire^35^.

#### Social interaction

The frequency of social interaction was assessed daily via a brief questionnaire developed by the research team. Participants were asked to report whether they had engaged in social interaction with (a) close contacts (immediate family, partner, close friends); (b) intermediate contacts (friends, relatives, colleagues); and (c) distant contacts (acquaintances, neighbours, strangers) over the past 24-h. Social interaction across all mediums were captured for each social group (face-to-face, video chat, phone chat, text messaging, email), with the total frequency of social interaction per social group for each individual being the sum of interaction mediums endorsed.

#### Physical isolation

The level of physical isolation was assessed daily via a brief questionnaire developed by the research team. Participants were asked to report whether they had been outside of their accommodation in the past 24 h for (a) work, (b) social purposes; (c) exercise/a walk; (d) shopping; (e) medical appointments; or (f) other. A participant’s daily physical isolation score was computed by summing the number of inhabitants they lived with plus the number of activities they engaged in outside their accommodation.

#### Depression

Depression level was assessed weekly using the Hospital Anxiety and Depression Scale (HADS)^36^. The depression subscale comprises seven items asking participants the degree to which they experienced emotional, cognitive, and motivational symptoms of depression on a scale of 0–3. Studies on large general community samples indicate that a score of 6 and above represents the top 25% of the distribution in terms of depression severity^37^. The cut-off score of 6 was therefore used in the present sample, with individuals scoring below 6 being labelled “low depression”, and above 6 being labelled “high depression” individuals. The HADS has demonstrated internal consistency and discriminant validity for assessing depression and anxiety symptom severity and case-ness, in both clinical populations as well as general populations^38^.

#### Demographic and contextual variables

##### Demographic questionnaire

Participants reported their age, gender, ethnic background, education level, employment status, disability status, and whether they have a current or past mental health condition diagnosis via a demographic questionnaire.

##### COVID-19 living context questionnaire

To capture the context in which participants were engaging in physically distanced living, a COVID-19 living context questionnaire was developed by the research team to assess a) the number of co-inhabits the participant is sharing their accommodation with; b) the number of days the participant has been engaging in physically distanced living prior to study commencement; including whether the participant had undergone any periods of strict isolation/quarantine prior to study commencement.

##### Anxiety

Due to its co-morbidity with depression, anxiety was also assessed to determine whether the effects of depression are unique to depression or general to emotional psychopathology. Anxiety was assessed alongside depression in the Hospital Anxiety and Depression Scale, where the anxiety subscale comprises seven items asking participants the degree to which they experienced cognitive and physical symptoms of anxiety on a scale of 0 to 3.

### Analyses plan

For hypothesis testing, mixed-effects regression modelling was conducted using the R packages *lme4*^39^ and *lmerTest*^40^. To determine whether social interaction frequency with each social group was uniquely related to loneliness over time, and whether such relationships are moderated by each of the three hypothesised moderators (Physical Isolation, Age, Depression), three separate models were fitted to test for a three-way interaction between the fixed factors of Assessment Point (Baseline week, Week 1, Week 2), Social Interaction Frequency (daily average), and each moderator. In addition, each model included independent fixed effects of each moderator variable as well as Duration of Physically Distanced living, Gender, Anxiety, General Mental Health Diagnosis Status. Each model was also fitted a random intercept of Subject and a random slope of Assessment Point.

#### Computation of weekly average scores

Social Interaction Frequency and Physical Isolation Level were assessed daily, and therefore required computation of daily averages for each of the three weeks of the study (Baseline week, Week 1, Week 2). While participants reported estimated daily averages for the Baseline week, Week 1 and Week 2 scores were computed by averaging the daily scores for that respective week.

#### Missing data handling

The proportion of data missing from key assessments was as follows; Thwarted Belongingness Scale items, 10.7%; physical isolation questionnaire items, 2.0%; and social interaction questionnaire items, 2.6%. Missing data was assumed to be missing at random. Given prior research demonstrating the superiority of multiple imputation over listwise deletion^41–43^, the present study imputed missing data via multilevel joint modelling multiple imputation using the R package *Jomo*^44^, using 20,000 burn-in iterations across 500 between-imputation iterations. Ten datasets were imputed following recommendations by White et al.^45^. Model convergence was achieved as indicated by an Ȓ value being close to 1 for all parameters^46^. Parameter estimates and inferences from the 10 imputed datasets were pooled using the R packages *mitml* and *pan*^47^.

## Results

### Participant characteristics

Demographics, physical distancing history, and mental health characteristics of the participant sample are presented in Table 1.

**Table 1:** Demographic characteristics of participants, N = 469.

Measure	Statistic
Age; *M* (*SD*), range	53.23 (15.16), 18–92
Gender; *N* (%)	Men = 97 (21%) Women = 372 (79%)
Living alone status (N)	Living alone = 98 Not living along = 371
Number of co-inhabitants; *M* (*SD*), range	1.41 (1.25), 0–8
Nominated cultural background; N	Australian = 326 Northern/Western European = 62 South-East Asian = 16 New Zealander = 15 North American = 11 Southern/Eastern European = 9 Southern-Central Asian = 4 South American = 3 Australian Aboriginal / Torres Strait Islander = 2 North-East Asian = 2 Other = 19
Highest education attainment; N	Less than high school = 19 High school = 74 Technical School = 92 University bachelor’s degree = 166 University postgraduate degree = 117 Missing = 1
Employment; N	Student = 15 Not employed = 76 Employed – part time = 92 Employed – full time = 130 Retired = 156 If employed: Working from home some days = 36 Working from home all days = 102
Mental health status, ‘ever diagnosed’; N (%)	No = 275 (58.7%) Yes—Past diagnosis = 113 (24%) Yes—Current diagnosis = 75 (16%) Not sure = 6 (1.3%)
Disability status; N	No = 440 Yes: Mild activity limitation = 19 Moderate activity limitation = 8 Severe activity limitation = 2
Days-ago physical distancing started; *M* (*SD*), range	49.57 (15.41), 0–112
Undergone period of self-isolation/quarantine prior to study commencement; N	Yes = 70 (15%) No = 397 (85%) Missing = 2
Number of days of self-isolation/quarantine, *M* (*SD*), Range	2.80 (8.50), 0–60

Descriptive statistics for non-demographic variables measured at baseline, and at each assessment point, are reported in Table 2.

**Table 2:** Descriptive statistics of baselines and weekly measures across participants, for each assessment point.

Measure	Assessment point								
	Day 0 (baseline)			Day 7			Day 14		
	M	SD	Range	M	SD	Range	M	SD	Range
***Baseline measures***									
HADS—depression Score	4.19	3.56	0–20						
HADS—anxiety Score	5.77	3.86	0–20						
***Weekly measures***									
Loneliness	16.12	9.59	7–48	16.12	9.73	7–49	15.63	9.95	7–49
Physical isolation (lower = more isolated)	2.82	1.57	0–10	2.23	1.28	0.17–8.83	2.22	1.29	0- 9.0
Social activity freq.—close contacts	3.36	1.29	0–5	1.66	0.90	0–4.57	1.72	0.96	0–4.8
Social activity freq.—intermediate contacts	2.91	1.35	0–5	1.46	0.97	0–4.5	1.55	1.04	0–4.75
Social activity freq.—distant contacts	1.88	1.34	0–5	1.06	0.80	0–4.67	1.17	0.90	0—5.0

The internal reliability of questionnaire measures completed by the present sample was evaluated by computing Cronbach’s alpha (*α*). Internal consistency of the Thwarted Belongingness Scale across the present study sample was *α* = 0.93. The internal consistency of the Depression subscale was *α* = 0.83 and the Anxiety subscale was *α* = 0.87.

### Assessing the relationship between loneliness and social interaction frequency

To test Hypothesis 1, the independent associations between social interaction frequency of close, intermediate, and distant contacts, and loneliness were each tested while statistically controlling for the effects of demographic, contextual, and mental health factors upon loneliness. Results from the mixed-effects model showed a significant independent relationship between Loneliness and Social Activity Frequency with Close Contacts, *b* = − 0.385, *p* = 0.013, C.I._95%_ = [− 0.688; − 0.082], but not between Loneliness and Social Activity Frequency with either Intermediate Contacts, *b* = − 0.096, *p* = 0.544, C.I._95%_ = [− 0.408; 0.215], or with Distant Contacts, *b* = 0.263, *p* = 0.086, C.I._95%_ = [− 0.038; 0.564]. These results indicated that greater social interaction frequency with close contacts was associated with lower loneliness, but social interaction frequency with intermediate or distant contacts was not associated with lower loneliness.

In regard to the other predictors in the model, an independent effect of Assessment Point was found, *b* = − 0.068, *p* = 0.026, C.I._95%_ = [− 0.128; − 0.008], where loneliness decreased overall with time. In addition, an independent effect of Age was found, *b* = − 0.049, *p* = 0.031, C.I._95%_ = [− 0.094; − 0.005], with younger age associated with higher Loneliness score. An independent effect of depression and of anxiety were also found, with higher Depression score associated with higher Loneliness score, *b* = 1.295, *p* < 0.001, C.I._95%_ = [1.069; 1.522], and higher Anxiety score associated with higher Loneliness score, *b* = 0.049, *p* < 0.001, C.I._95%_ = [0.274; 0.705]. Mental health diagnosis status was also independently related to loneliness, where compared to participants without a diagnosis, participants with a current diagnosis, *b* = 2.178, *p* = 0.023, C.I._95%_ = [0.294; 4.062], past diagnosis, *b* = 1.915, *p* = 0.012, C.I._95%_ = [0.415; 3.415], or who were unsure if they have ever had a diagnosis, *b* = 8.822, *p* = 0.002, C.I._95%_ = [3.34; 14.31], reporting higher Loneliness scores. Effect estimates arising from this model are reported in Table S1.

Therefore, younger age or higher depression level were associated with greater loneliness over the study period. Of most importance, these results indicated that greater social interaction frequency with close contacts, but not intermediate or distant contacts, was associated with lower loneliness. Thus, the results supported Hypothesis 1.

### Moderation analyses

As described in the analyses plan, separate models were fitted for social interaction with Close Contacts, Intermediate Contacts, and Distant Contacts to investigate the presence of interaction effects of Assessment Point, Social Interaction Frequency and the hypothesized moderator variables of Physical Isolation, Age, and Depression. The results of each tested model, for each hypothesized moderator variable, are now described in turn.

#### Moderating role of physical isolation

In the model fitting Social Interaction Frequency with Close Contacts in an interaction term with Assessment Point and Physical Isolation, results did not reveal a significant three-way interaction, *b* = 0.013, *p* = 0.432, C.I._95%_ = [− 0.020; 046]. Results also did not reveal any significant two-way interactions involving these three variables. Effect estimates arising from this model are reported in Table S2.

In the model fitting Social Interaction Frequency with Intermediate Contacts in an interaction term with Assessment Point and Physical Isolation, results did not reveal a significant three-way interaction, *b* = 0.009, *p* = 0.559, C.I._95%_ = [− 0.022; 040]**,** nor any significant two-way interactions involving these three variables. Effect estimates arising from this model are reported in Table S3.

In the model fitting Social Interaction Frequency with Distant Contacts in an interaction term with Assessment Point and Physical Isolation, results did not reveal a significant three-way interaction, *b* = − 0.001, *p* = 0.946, C.I._95%_ = [− 0.039; 0.037] nor any significant two-way interactions involving these three variables. Effect estimates arising from this model are reported in Table S4.

Thus, the results of these analyses indicated that Physical Isolation did not significantly moderate the relationship between Social Interaction Frequency and Loneliness, irrespective of social contact category. Thus, the results of these analyses provided no support for Hypothesis 2.

#### Moderating role of age

The model fitting Social Interaction Frequency with Close Contacts in an interaction term with Assessment Point and Age did not reveal a significant three-way interaction, *b* = − 0.000, *p* = 0.920, C.I._95%_ = [− 0.003; 0.003], nor any significant two-way interactions involving these three variables. Effect estimates arising from this model are reported in Table S5.

The model fitting Social Interaction Frequency with Intermediate Contacts in an interaction term with Assessment Point and Age did not reveal a significant three-way interaction, *b* = − 0.001, *p* = 0.349, C.I._95%_ = [− 0.004; 0.001], nor any significant two-way interactions involving these three variables. Effect estimates arising from this model are reported in Table S6.

The model fitting Social Interaction Frequency with Distant Contacts in an interaction term with Assessment Point and Age did not reveal a significant three-way interaction, *b* = 0.000, *p* = 0.860, C.I._95%_ = [− 0.003; 0.003], nor any significant two-way interactions involving these three variables. Effect estimates arising from this model are reported in Table S7.

Thus, the results of these analyses indicated that Age did not significantly moderate the relationship between Social Interaction Frequency and Loneliness, irrespective of social contact category. Thus, the results of these analyses provided no support for Hypothesis 3.

#### Moderating role of depression

In the model fitting Social Interaction Frequency with Close Contacts in an interaction term with Assessment Point and Depression, results revealed a significant three-way interaction, *b* = − 0.014, *p* = 0.025, C.I._95%_ = [− 0.026; − 0.002]. Effect estimates arising from this model are reported in Table S8.

Next, the model fitting Social Interaction Frequency with Intermediate Contacts in an interaction term with Assessment Point and Depression did not reveal a significant three-way interaction, *b* = − 0.008, *p* = 0.214, C.I._95%_ = [− 0.020; 0.004], nor any significant two-way interactions involving these three variables. Effect estimates arising from this model are reported in Table S10.

The model fitting Social Interaction Frequency with Distant Contacts in an interaction term with Assessment Point and Depression did not revealed a significant three-way interaction, *b* = − 0.013, *p* = 0.072, C.I._95%_ = [− 0.026; 0.001], nor any significant two-way interactions involving these three variables. Effect estimates arising from this model are reported in Table S11.

**Figure 1:**
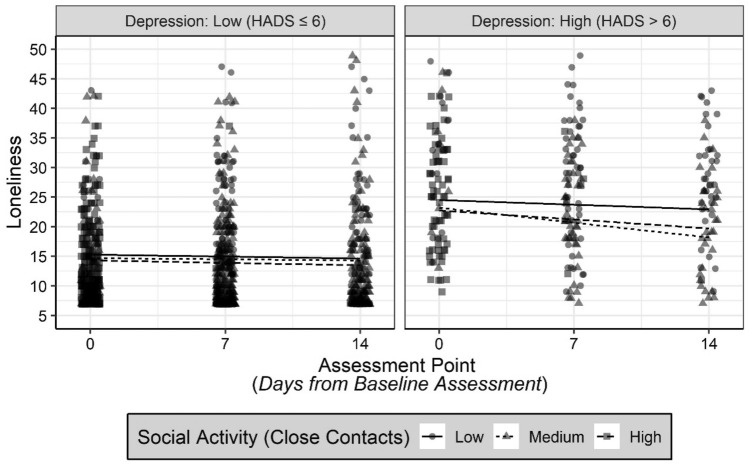
Visualisation of data drawn from one randomly selected imputed dataset depicting the relationship between social interaction frequency with close contacts and loneliness, at each assessment point for participants in the high depression group and low depression group.

To further delineate the nature of the three-way interaction between social activity with close contacts, loneliness, and depression, a post-hoc decomposition of this three-way interaction examined depression level as a categorical variable. Grouping was based on clinical cut-off scores^38^ that classed a Low Depression group with HADS score ≤ 6 (N = 375), and a High Depression group with HADS score > 6 (N = 94). The results of the analysis showed a significant two-way interaction effect involving Social Interaction Frequency with Close Contacts and Assessment Point significantly predicted Loneliness amongst participants in the High Depressing group, *b* = − 0.129, *p* = 0.041, C.I._95%_ = [− 0.252; − 005], but not amongst participants in the Low Depression group *b* = 0.00, *p* = 0.98, C.I._95%_ = [− 0.049; 048]. Effect estimates arising from each of these post-hoc models are reported in Table S9a,b, respectively. Visualisation of data drawn from one randomly selected imputed dataset depicting the relationship between Social Interaction Frequency with Close Contacts and Loneliness, at each Assessment Point for participants in the High Depression group and Low Depression group, is presented in Fig. 1.

Thus, the results of these analyses indicated that Depression significantly moderated the relationship between Social Interaction Frequency and Loneliness when considering Close Contacts, but not Intermediate Contacts or Distant Contacts, providing support for Hypothesis 4.

## Discussion

The present study investigated the relationship between levels of loneliness and the frequency of social interaction with close, intermediate, and distant social contacts over two-weeks of physical distancing, and whether these relationships were moderated by physical isolation level, age, or depression.

The findings indicate that more frequent social interaction with close contacts, but not with intermediate or distant contacts, was associated with lower overall loneliness. Furthermore, depression was found to be a significant moderator of this relationship, such that more frequent social interaction with close contacts was associated with a decline in loneliness over the assessment period, but only for individuals who reported high levels of depression symptoms. In contrast, the frequency of social interaction with close contacts had no effect on loneliness amongst individuals who reported lower levels of depression symptoms.

In addition, having higher anxiety, having a previous or current mental health diagnosis, and being of younger age were associated with greater levels of loneliness in general over the assessed period. However, age did not moderate the relationship between social interaction frequency with close contacts and loneliness. In contrast, level of physical isolation from others did not predict level of loneliness, nor did it moderate the relationship between social interaction frequency with close contacts and loneliness.

### Implications

Importantly, the present study is the first to show that modifiable behavioural factors, such as social interaction with close contacts, may serve to increase resilience to loneliness irrespective of one’s physical isolation level or age. Such findings indicate that engagement in interaction with specifically close social contacts may serve to reduce elevated loneliness across individuals during periods of locked-down living. Further, the present findings indicate that the protective effects of interacting with close social contacts to reduce the risk of loneliness may be particularly beneficial for individuals experiencing elevated depression symptoms, whose levels of loneliness were found to be more strongly predicted by lower frequency of social interactions with close contacts than was the case for people low in depression symptoms. Together, these findings indicate that promoting social interaction with close social contacts, such as family and close friends, could serve as an avenue for reducing the risk of elevated loneliness during periods of locked-down living for individuals experiencing depression symptoms.

### Relation to previous research

The results of the present study align with previous research that has also identified increased use of digital communication technology for social interaction to be associated with reduced levels of loneliness during periods of physical isolation^48^, and so provides additional support for the benefits of digital technologies in maintaining mental health during lockdowns. Similarly, the present findings are consistent with research indicating the benefits of access to digital communication devices and internet during periods of quarantine, a fundamental means of social interaction during physical isolation. Previous research has identified lack of access to digital communication devices and internet to be associated with elevated mental health burden during periods of community quarantine^3^, and researchers have identified digital inequality to as a significant challenge to population level mental well-being during period of community physical isolation^49^. The present findings support such conclusions by indicating the protective benefits to loneliness of online social communication during physical isolation.

The present finding that younger age and severity of pre-existing depression symptoms is associated with greater elevation in loneliness during physical isolation is consistent with findings of numerous studies the have revealed individual characterises predictive of elevated loneliness and diminished mental health resulting from physical isolation during the COVID-19 pandemic. For example, cross-sectional studies conducted during a similar time period in the United Kingdom have also identified demographics factors such as younger age, the presence of depression or anxiety symptoms, or having a present or past mental health condition, to be risk factors for greater levels of loneliness^12,28^. Likewise, younger age, female gender, and poorer financial status have been identified to be determinants of elevated COVID-19 related anxiety among Filipino college students^3^, and younger age was associated with greater loneliness amongst Italian residents^48^.

Some findings of the present study contrast with previous findings. For example, in contrast to Groarke et al.^28^ the present study did not find level of physical isolation, operationalised as lower numbers of co-inhabitants and of interactions outside the home, to be a risk factor for elevated loneliness. This discrepant finding may be due to contextual differences between Australia and the UK, such as the overall severity of COVID-19 impacts (deaths and hospitalisations), the perceived effectiveness of government policies in relation to containing COVID-19^50^, or the relative size of dwellings and population density^51^.

### Limitations

It is important to consider the interpretation of findings from the present study in the light of several limitations. First, the present study recruited a self-selected convenience sample that cannot be assumed to be a representative cross-section of the general population. As such, it remains to be determined whether the findings demonstrated in the present study would be replicated in a stratified sample of the general population. Second, the study was conducted over 14-days, and therefore the associations between the assessed variables across a longer-term are unknown. Third, the present findings reflect experiences during an initial period of locked-down living in response to the pandemic, and it remains to be tested whether social interaction with close contacts remain beneficial in promoting resilience to loneliness amongst individuals who have repeated experiences of locked-down living.

### Conclusion

During times of elevated objective physical isolation, such as due to global pandemics, it is critical to understand who is most vulnerable to experiencing elevated loneliness, and how modifiable behavioural factors, such as social interaction, can promote resilience against loneliness. The present findings indicate that promotion of frequent social interactions with close family and friends may buffer against loneliness across individuals generally, and amongst those experiencing depression symptoms specifically.

### Supplementary Information

Supplementary Tables.

## Data Availability

The study’s Open Science Framework can be found at https://osf.io/yfz3n/.
