# Author Correction: Social activity promotes resilience against loneliness in depressed individuals: a study over 14 days of physical isolation during the COVID-19 pandemic in Australia

**DOI:** 10.1038/s41598-024-53556-5

**Published:** 2024-02-07

**Authors:** Julie L. Ji, Julian Basanovic, Colin MacLeod

**Affiliations:** School of Psychological Science, University of Western Australia, M034 35 Stirling Highway, Perth, WA 6009 Australia

Correction to: *Scientific Reports* 10.1038/s41598-022-11315-4, published online 03 May 2022

The original version of this Article contained errors.

In the original version of this Article, the *p* values in the Results section were swapped.

“Results from the mixed-effects model showed a significant independent relationship between Loneliness and Social Activity Frequency with Close Contacts, *b* = − 0.385, *p* = 0.544, C.I._95%_ = [− 0.688; − 0.082], but not between Loneliness and Social Activity Frequency with either Intermediate Contacts, *b* = − 0.096, *p* = 0.086, C.I._95%_ = [− 0.408; 0.215], or with Distant Contacts, *b* = 0.263, *p* = 0.013, C.I._95%_ = [− 0.038; 0.564]. These results indicated that greater social interaction frequency with close contacts was associated with lower loneliness, but social interaction frequency with intermediate or distant contacts was not associated with lower loneliness.”

now reads,

“Results from the mixed-effects model showed a significant independent relationship between Loneliness and Social Activity Frequency with Close Contacts, *b* = − 0.385, *p* = 0.013, C.I._95%_ = [− 0.688; − 0.082], but not between Loneliness and Social Activity Frequency with either Intermediate Contacts, *b* = − 0.096, *p* = 0.544, C.I._95%_ = [− 0.408; 0.215], or with Distant Contacts, *b* = 0.263, *p* = 0.086, C.I._95%_ = [− 0.038; 0.564]. These results indicated that greater social interaction frequency with close contacts was associated with lower loneliness, but social interaction frequency with intermediate or distant contacts was not associated with lower loneliness.”

In addition, the Data Availability section was omitted. It now reads,

“Data availability

The study’s Open Science Framework can be found at https://osf.io/yfz3n/.”

The original Article has been corrected.

