# Supplementary material for: Social activity promotes resilience against loneliness in depressed individuals: a study over 14-days of physical isolation during the COVID-19 pandemic in Australia
Source: Sci Rep. 2022 May 3;12:7155. doi: 10.1038/s41598-022-11315-4 (PMC9062834; doi:10.1038/s41598-022-11315-4)
Supplement: Supplementary file 1 — Supplementary Tables. [file 41598_2022_11315_MOESM1_ESM.docx]

Table S1: Effect estimates from imputed models examining the impact of predictor variables on Loneliness scores.

| **Predictor** | **Estimate** | **2.5 %** | **97.5 %** | ***SE*** | ***t*** | **df** | ***p*** |
| --- | --- | --- | --- | --- | --- | --- | --- |
| (Intercept) | 12.813 | 9.037 | 16.59 | 1.927 | 6.65 | 31120.415 | **< .001** |
| Assessment Point (Days from Baseline assessment) | -0.068 | -0.128 | -0.008 | 0.03 | -2.264 | 83.96 | **0.026** |
| Social Activity Freq. - Close Contacts | -0.385 | -0.688 | -0.082 | 0.154 | -2.495 | 712.793 | **0.013** |
| Physical Isolation (lower = more isolated) | -0.153 | -0.488 | 0.183 | 0.171 | -0.891 | 15758.166 | 0.373 |
| Social Activity Freq. - Intermediate Contacts | -0.096 | -0.408 | 0.215 | 0.159 | -0.608 | 599.467 | 0.544 |
| Social Activity Freq. - Distant Contacts | 0.263 | -0.038 | 0.564 | 0.153 | 1.715 | 5593.182 | **0.086** |
| Days in physical isolation | -0.053 | -0.126 | 0.02 | 0.037 | -1.432 | 32211160.895 | 0.152 |
| HADS - Depression Score | 1.295 | 1.069 | 1.522 | 0.116 | 11.196 | 161116.653 | **< .001** |
| Mental Health Status (‘Not sure’) ^^^ | 8.822 | 3.335 | 14.309 | 2.799 | 3.151 | 278693378.427 | **0.002** |
| Mental Health Status (Current Diagnosis) ^^^ | 2.178 | 0.295 | 4.062 | 0.961 | 2.267 | 608930.247 | **0.023** |
| Mental Health Status (Past Diagnosis) ^^^ | 1.915 | 0.415 | 3.415 | 0.765 | 2.502 | 13910762.584 | **0.012** |
| HADS - Anxiety Score | 0.49 | 0.274 | 0.705 | 0.11 | 4.455 | 4524035.075 | **< .001** |
| Age (years) | -0.049 | -0.094 | -0.005 | 0.023 | -2.16 | 3447552.198 | **0.031** |
| Gender (Ref* Male) | 0.047 | -1.509 | 1.603 | 0.794 | 0.059 | 2334432.667 | 0.953 |
| Distancing Duration (Days Practicing) | -0.034 | -0.075 | 0.007 | 0.021 | -1.617 | 17050841.056 | 0.106 |

Note: ^^^ Mental Health Status variables computed relative to reference response level ‘No’.

Table S2: Effect estimates from imputed models examining the impact of predictor variables on Loneliness scores.

| **Predictor** | **Estimate** | **2.5 %** | **97.5 %** | ***SE*** | ***t*** | **df** | ***p*** |
| --- | --- | --- | --- | --- | --- | --- | --- |
| (Intercept) | 12.592 | 8.5 | 16.685 | 2.088 | 6.031 | 13883.485 | **< .001** |
| Assessment Point (Days from Baseline assessment) | 0.054 | -0.145 | 0.252 | 0.101 | 0.532 | 286.064 | 0.595 |
| Social Activity Freq. - Close Contacts | -0.299 | -0.884 | 0.286 | 0.298 | -1.003 | 1452.352 | 0.316 |
| Physical Isolation (lower = more isolated) | -0.083 | -0.753 | 0.587 | 0.342 | -0.242 | 5100.379 | 0.809 |
| Social Activity Freq. - Intermediate Contacts | -0.104 | -0.418 | 0.21 | 0.16 | -0.653 | 535.004 | 0.514 |
| Social Activity Freq. - Distant Contacts | 0.265 | -0.036 | 0.566 | 0.154 | 1.725 | 5252.359 | 0.085 |
| Days in physical isolation | -0.053 | -0.125 | 0.02 | 0.037 | -1.424 | 18196159.322 | 0.154 |
| HADS - Depression Score | 1.292 | 1.065 | 1.519 | 0.116 | 11.162 | 115442.914 | **< .001** |
| Mental Health Status (‘Not sure’) ^^^ | 8.803 | 3.317 | 14.289 | 2.799 | 3.145 | 190386584.118 | **0.002** |
| Mental Health Status (Current Diagnosis) ^^^ | 2.159 | 0.275 | 4.043 | 0.961 | 2.246 | 574351.359 | **0.025** |
| Mental Health Status (Past Diagnosis) ^^^ | 1.914 | 0.413 | 3.414 | 0.765 | 2.5 | 18507001.834 | **0.012** |
| HADS - Anxiety Score | 0.492 | 0.276 | 0.707 | 0.11 | 4.474 | 4527239.401 | **< .001** |
| Age (years) | -0.05 | -0.095 | -0.006 | 0.023 | -2.202 | 4014406.185 | **0.028** |
| Gender (Ref: Male) | 0.053 | -1.503 | 1.609 | 0.794 | 0.067 | 1969672.246 | 0.947 |
| Distancing Duration (Days Practicing) | -0.034 | -0.075 | 0.007 | 0.021 | -1.619 | 5216757.901 | 0.105 |
| Assessment Point*Social Activity Freq. - Close Contacts | -0.046 | -0.132 | 0.041 | 0.044 | -1.041 | 156.184 | 0.299 |
| Assessment Point*Physical Isolation | -0.04 | -0.115 | 0.034 | 0.038 | -1.064 | 383.819 | 0.288 |
| Social Activity Freq. - Close Contacts*Physical Isolation | -0.018 | -0.193 | 0.157 | 0.089 | -0.205 | 5686.52 | 0.838 |
| Assessment Point*Social Activity Freq. - Close Contacts*Physical Isolation | 0.013 | -0.02 | 0.046 | 0.017 | 0.788 | 134.631 | 0.432 |

Note: ^^^ Mental Health Status variables computed relative to reference response level ‘No’.

Table S3: Effect estimates from imputed models examining the impact of predictor variables on Loneliness scores.

| **Predictor** | **Estimate** | **2.5 %** | **97.5 %** | ***SE*** | ***t*** | **df** | ***p*** |
| --- | --- | --- | --- | --- | --- | --- | --- |
| (Intercept) | 12.093 | 8.112 | 16.075 | 2.031 | 5.954 | 11823.656 | **< .001** |
| Assessment Point (Days from Baseline assessment) | 0.041 | -0.118 | 0.201 | 0.081 | 0.507 | 1817.123 | 0.612 |
| Social Activity Freq. - Intermediate Contacts | 0.174 | -0.378 | 0.726 | 0.281 | 0.618 | 2303.75 | 0.537 |
| Physical Isolation (lower = more isolated) | 0.143 | -0.466 | 0.752 | 0.31 | 0.461 | 2469.108 | 0.645 |
| Social Activity Freq. - Close Contacts | -0.378 | -0.68 | -0.076 | 0.154 | -2.454 | 772.391 | **0.014** |
| Social Activity Freq. - Distant Contacts | 0.266 | -0.035 | 0.567 | 0.153 | 1.733 | 5071.698 | 0.083 |
| Days in physical isolation | -0.054 | -0.127 | 0.019 | 0.037 | -1.454 | 53574237.58 | 0.146 |
| HADS - Depression Score | 1.29 | 1.063 | 1.517 | 0.116 | 11.136 | 216078.81 | **< .001** |
| Mental Health Status (‘Not sure’) ^^^ | 8.832 | 3.337 | 14.326 | 2.803 | 3.15 | 205623376.009 | **0.002** |
| Mental Health Status (Current Diagnosis) ^^^ | 2.146 | 0.261 | 4.032 | 0.962 | 2.231 | 1025787.095 | **0.026** |
| Mental Health Status (Past Diagnosis) ^^^ | 1.893 | 0.39 | 3.396 | 0.767 | 2.469 | 15723907.844 | **0.014** |
| HADS - Anxiety Score | 0.493 | 0.277 | 0.708 | 0.11 | 4.476 | 5447801.726 | **< .001** |
| Age (years) | -0.05 | -0.095 | -0.005 | 0.023 | -2.181 | 2683395.956 | **0.029** |
| Gender (Ref: Male) | 0.047 | -1.511 | 1.604 | 0.795 | 0.059 | 3912839.649 | 0.953 |
| Distancing Duration (Days Practicing) | -0.034 | -0.075 | 0.007 | 0.021 | -1.649 | 6165557.55 | 0.099 |
| Assessment Point*Social Activity Freq. - Intermediate Contacts | -0.032 | -0.111 | 0.047 | 0.04 | -0.801 | 205.527 | 0.424 |
| Assessment Point*Physical Isolation | -0.039 | -0.098 | 0.02 | 0.03 | -1.287 | 1094.473 | 0.198 |
| Social Activity Freq. - Intermediate Contacts*Physical Isolation | -0.1 | -0.28 | 0.08 | 0.092 | -1.092 | 1911.213 | 0.275 |
| Assessment Point*Social Activity Freq. - Intermediate Contacts*Physical Isolation | 0.009 | -0.022 | 0.04 | 0.016 | 0.586 | 118.572 | 0.559 |

Note: ^^^ Mental Health Status variables computed relative to reference response level ‘No’.

Table S4: Effect estimates from imputed models examining the impact of predictor variables on Loneliness scores.

| **Predictor** | **Estimate** | **2.5 %** | **97.5 %** | ***SE*** | ***t*** | **df** | ***p*** |
| --- | --- | --- | --- | --- | --- | --- | --- |
| (Intercept) | 12.759 | 8.864 | 16.655 | 1.987 | 6.42 | 18460.105 | **< .001** |
| Assessment Point (Days from Baseline assessment) | -0.04 | -0.205 | 0.125 | 0.083 | -0.475 | 111.921 | 0.636 |
| Social Activity Freq. - Distant Contacts | 0.283 | -0.363 | 0.929 | 0.329 | 0.859 | 3644.374 | 0.39 |
| Physical Isolation (lower = more isolated) | -0.115 | -0.591 | 0.36 | 0.243 | -0.474 | 41648.786 | 0.635 |
| Social Activity Freq. - Close Contacts | -0.388 | -0.693 | -0.084 | 0.155 | -2.503 | 643.471 | **0.013** |
| Social Activity Freq. - Intermediate Contacts | -0.101 | -0.413 | 0.211 | 0.159 | -0.635 | 609.815 | 0.526 |
| Days in physical isolation | -0.053 | -0.125 | 0.02 | 0.037 | -1.421 | 18141795.055 | 0.155 |
| HADS - Depression Score | 1.295 | 1.068 | 1.521 | 0.116 | 11.189 | 197062.8 | **< .001** |
| Mental Health Status (‘Not sure’) ^^^ | 8.804 | 3.316 | 14.291 | 2.8 | 3.145 | 242629484.488 | **0.002** |
| Mental Health Status (Current Diagnosis) ^^^ | 2.165 | 0.282 | 4.049 | 0.961 | 2.253 | 871320.829 | **0.024** |
| Mental Health Status (Past Diagnosis) ^^^ | 1.906 | 0.404 | 3.408 | 0.766 | 2.487 | 17700276.724 | **0.013** |
| HADS - Anxiety Score | 0.491 | 0.275 | 0.706 | 0.11 | 4.465 | 5018255.043 | **< .001** |
| Age (years) | -0.05 | -0.095 | -0.005 | 0.023 | -2.181 | 4961726.436 | **0.029** |
| Gender (Ref: Male) | 0.043 | -1.513 | 1.599 | 0.794 | 0.054 | 2934178.366 | 0.957 |
| Distancing Duration (Days Practicing) | -0.034 | -0.074 | 0.007 | 0.021 | -1.61 | 10516169.267 | 0.107 |
| Assessment Point*Social Activity Freq. - Distant Contacts | 0.007 | -0.096 | 0.109 | 0.051 | 0.127 | 97.379 | 0.899 |
| Assessment Point*Physical Isolation | -0.015 | -0.072 | 0.043 | 0.029 | -0.502 | 161.759 | 0.616 |
| Social Activity Freq. - Distant Contacts*Physical Isolation | -0.009 | -0.198 | 0.18 | 0.096 | -0.091 | 12171.93 | 0.927 |
| Assessment Point*Social Activity Freq. - Distant Contacts*Physical Isolation | -0.001 | -0.039 | 0.037 | 0.019 | -0.068 | 76.4 | 0.946 |

Note: ^^^ Mental Health Status variables computed relative to reference response level ‘No’.

Table S5: Effect estimates from imputed models examining the impact of predictor variables on Loneliness scores.

| **Predictor** | **Estimate** | **2.5 %** | **97.5 %** | ***SE*** | ***t*** | **df** | ***p*** |
| --- | --- | --- | --- | --- | --- | --- | --- |
| (Intercept) | 12.775 | 8.013 | 17.536 | 2.429 | 5.26 | 4074.789 | **< .001** |
| Assessment Point (Days from Baseline assessment) | -0.045 | -0.411 | 0.321 | 0.186 | -0.241 | 542.383 | 0.81 |
| Social Activity Freq. - Close Contacts | -0.345 | -1.374 | 0.684 | 0.524 | -0.658 | 1363.355 | 0.51 |
| Age (years) | -0.051 | -0.121 | 0.019 | 0.036 | -1.42 | 3194.52 | 0.156 |
| Social Activity Freq. - Intermediate Contacts | -0.102 | -0.415 | 0.212 | 0.16 | -0.637 | 548.095 | 0.524 |
| Social Activity Freq. - Distant Contacts | 0.26 | -0.041 | 0.561 | 0.154 | 1.694 | 6105.731 | 0.09 |
| Physical Isolation (lower = more isolated) | -0.164 | -0.501 | 0.173 | 0.172 | -0.955 | 14814.28 | 0.34 |
| HADS - Anxiety Score | 0.49 | 0.275 | 0.706 | 0.11 | 4.458 | 3572315.777 | **< .001** |
| HADS - Depression Score | 1.293 | 1.066 | 1.52 | 0.116 | 11.168 | 141498.147 | **< .001** |
| Gender (Ref: Male) | 0.054 | -1.502 | 1.61 | 0.794 | 0.067 | 2319967.959 | 0.946 |
| Days in physical isolation | -0.053 | -0.126 | 0.02 | 0.037 | -1.424 | 10738869.842 | 0.154 |
| Mental Health Status (‘Not sure’) ^^^ | 8.82 | 3.331 | 14.308 | 2.8 | 3.149 | 114702520.868 | **0.002** |
| Mental Health Status (Current Diagnosis) ^^^ | 2.182 | 0.298 | 4.067 | 0.961 | 2.27 | 471522.47 | **0.023** |
| Mental Health Status (Past Diagnosis) ^^^ | 1.923 | 0.423 | 3.424 | 0.766 | 2.512 | 14430713.729 | **0.012** |
| Distancing Duration (Days Practicing) | -0.034 | -0.075 | 0.007 | 0.021 | -1.626 | 6670742.689 | 0.104 |
| Assessment Point*Social Activity Freq. - Close Contacts | -0.01 | -0.181 | 0.161 | 0.087 | -0.117 | 168.96 | 0.907 |
| Assessment Point*Age) | 0 | -0.006 | 0.007 | 0.003 | 0.073 | 1506.31 | 0.941 |
| Social Activity Freq. - Close Contacts*Age | 0 | -0.018 | 0.018 | 0.009 | 0.027 | 2130.12 | 0.978 |
| Assessment Point*Social Activity Freq. - Close Contacts*Age | 0 | -0.003 | 0.003 | 0.001 | -0.1 | 314.502 | 0.92 |

Note: ^^^ Mental Health Status variables computed relative to reference response level ‘No’.

Table S6: Effect estimates from imputed models examining the impact of predictor variables on Loneliness scores.

| **Predictor** | **Estimate** | **2.5 %** | **97.5 %** | ***SE*** | ***t*** | **df** | ***p*** |
| --- | --- | --- | --- | --- | --- | --- | --- |
| (Intercept) | 14.697 | 10.158 | 19.236 | 2.315 | 6.347 | 7113.157 | **< .001** |
| Assessment Point (Days from Baseline assessment) | -0.221 | -0.538 | 0.097 | 0.162 | -1.364 | 745.761 | 0.173 |
| Social Activity Freq. - Intermediate Contacts | -0.82 | -1.814 | 0.175 | 0.507 | -1.615 | 3797.507 | 0.106 |
| Age (years) | -0.085 | -0.15 | -0.021 | 0.033 | -2.589 | 5692.007 | **0.01** |
| Social Activity Freq. - Close Contacts | -0.385 | -0.688 | -0.081 | 0.155 | -2.49 | 681.43 | **0.013** |
| Social Activity Freq. - Distant Contacts | 0.252 | -0.049 | 0.553 | 0.154 | 1.641 | 5189.919 | 0.101 |
| Physical Isolation (lower = more isolated) | -0.148 | -0.485 | 0.189 | 0.172 | -0.86 | 12353.036 | 0.39 |
| HADS - Anxiety Score | 0.492 | 0.277 | 0.708 | 0.11 | 4.475 | 2871794.795 | **< .001** |
| HADS - Depression Score | 1.29 | 1.063 | 1.517 | 0.116 | 11.139 | 167287.168 | **< .001** |
| Gender (Ref: Male) | 0.043 | -1.513 | 1.6 | 0.794 | 0.055 | 4231889.412 | 0.956 |
| Days in physical isolation | -0.052 | -0.125 | 0.02 | 0.037 | -1.41 | 48812219.786 | 0.159 |
| Mental Health Status (‘Not sure’) ^^^ | 8.704 | 3.211 | 14.197 | 2.803 | 3.106 | 72290859.494 | **0.002** |
| Mental Health Status (Current Diagnosis) ^^^ | 2.214 | 0.33 | 4.099 | 0.962 | 2.303 | 670739.416 | **0.021** |
| Mental Health Status (Past Diagnosis) ^^^ | 1.925 | 0.424 | 3.426 | 0.766 | 2.513 | 22116957.79 | **0.012** |
| Distancing Duration (Days Practicing) | -0.034 | -0.075 | 0.007 | 0.021 | -1.638 | 8051426.266 | 0.101 |
| Assessment Point*Social Activity Freq. - Intermediate Contacts | 0.058 | -0.095 | 0.211 | 0.078 | 0.751 | 231.879 | 0.454 |
| Assessment Point*Age | 0.003 | -0.002 | 0.009 | 0.003 | 1.097 | 986.055 | 0.273 |
| Social Activity Freq. - Intermediate Contacts*Age | 0.014 | -0.004 | 0.031 | 0.009 | 1.559 | 4642.395 | 0.119 |
| Assessment Point*Social Activity Freq. - Intermediate Contacts*Age | -0.001 | -0.004 | 0.001 | 0.001 | -0.937 | 564.8 | 0.349 |

Note: ^^^ Mental Health Status variables computed relative to reference response level ‘No’.

Table S7: Effect estimates from imputed models examining the impact of predictor variables on Loneliness scores.

| **Predictor** | **Estimate** | **2.5 %** | **97.5 %** | ***SE*** | ***t*** | **df** | ***p*** |
| --- | --- | --- | --- | --- | --- | --- | --- |
| (Intercept) | 13.024 | 8.848 | 17.201 | 2.131 | 6.113 | 7394.543 | **< .001** |
| Assessment Point (Days from Baseline assessment) | -0.059 | -0.308 | 0.19 | 0.127 | -0.468 | 490.689 | 0.64 |
| Social Activity Freq. - Distant Contacts | 0.112 | -0.998 | 1.221 | 0.566 | 0.197 | 4001.112 | 0.844 |
| Age (years) | -0.053 | -0.109 | 0.003 | 0.029 | -1.852 | 7879.01 | 0.064 |
| Social Activity Freq. - Close Contacts | -0.389 | -0.693 | -0.086 | 0.155 | -2.517 | 721.258 | **0.012** |
| Social Activity Freq. - Intermediate Contacts | -0.097 | -0.409 | 0.215 | 0.159 | -0.614 | 599.671 | 0.54 |
| Physical Isolation (lower = more isolated) | -0.148 | -0.485 | 0.189 | 0.172 | -0.863 | 12194.244 | 0.388 |
| HADS - Anxiety Score | 0.491 | 0.275 | 0.706 | 0.11 | 4.465 | 4379385.355 | **< .001** |
| HADS - Depression Score | 1.294 | 1.067 | 1.521 | 0.116 | 11.18 | 193243.48 | **< .001** |
| Gender (Ref: Male) | 0.045 | -1.51 | 1.601 | 0.794 | 0.057 | 3582012.987 | 0.954 |
| Days in physical isolation | -0.053 | -0.126 | 0.019 | 0.037 | -1.437 | 14422185.79 | 0.151 |
| Mental Health Status (‘Not sure’) ^^^ | 8.848 | 3.359 | 14.338 | 2.801 | 3.159 | 271752491.741 | **0.002** |
| Mental Health Status (Current Diagnosis) ^^^ | 2.183 | 0.3 | 4.066 | 0.961 | 2.272 | 717081.077 | **0.023** |
| Mental Health Status (Past Diagnosis) ^^^ | 1.908 | 0.407 | 3.409 | 0.766 | 2.492 | 10856094.696 | **0.013** |
| Distancing Duration (Days Practicing) | -0.034 | -0.075 | 0.007 | 0.021 | -1.614 | 17892666.171 | 0.107 |
| Assessment Point*Social Activity Freq. - Distant Contacts | -0.012 | -0.172 | 0.149 | 0.082 | -0.141 | 1098.109 | 0.888 |
| Assessment Point*Age | 0 | -0.005 | 0.004 | 0.002 | -0.102 | 405.174 | 0.919 |
| Social Activity Freq. - Distant Contacts*Age | 0.003 | -0.018 | 0.023 | 0.01 | 0.259 | 2751.9 | 0.796 |
| Assessment Point*Social Activity Freq. - Distant Contacts*Age | 0 | -0.003 | 0.003 | 0.001 | 0.177 | 797.143 | 0.86 |

Note: ^^^ Mental Health Status variables computed relative to reference response level ‘No’.

Table S8: Effect estimates from imputed models examining the impact of predictor variables on Loneliness scores.

| **Predictor** | **Estimate** | **2.5 %** | **97.5 %** | ***SE*** | ***t*** | **df** | ***p*** |
| --- | --- | --- | --- | --- | --- | --- | --- |
| (Intercept) | 12.516 | 8.652 | 16.379 | 1.971 | 6.349 | 16707.875 | **< .001** |
| Assessment Point (Days from Baseline assessment) | -0.048 | -0.208 | 0.112 | 0.081 | -0.591 | 141.792 | 0.556 |
| Social Activity Freq. - Close Contacts | -0.29 | -0.744 | 0.164 | 0.232 | -1.251 | 1999.215 | 0.211 |
| HADS - Depression Score | 1.353 | 1.025 | 1.68 | 0.167 | 8.119 | 440.096 | **< .001** |
| Mental Health Status (‘Not sure’) ^^^ | 8.727 | 3.245 | 14.209 | 2.797 | 3.12 | 106019563.893 | **0.002** |
| Mental Health Status (Current Diagnosis) ^^^ | 2.202 | 0.321 | 4.083 | 0.96 | 2.295 | 977247.191 | **0.022** |
| Mental Health Status (Past Diagnosis) ^^^ | 1.955 | 0.456 | 3.454 | 0.765 | 2.556 | 16766924.058 | **0.011** |
| Social Activity Freq. - Intermediate Contacts | -0.095 | -0.405 | 0.216 | 0.158 | -0.6 | 580.473 | 0.549 |
| Social Activity Freq. - Distant Contacts | 0.279 | -0.02 | 0.579 | 0.153 | 1.826 | 4522.011 | 0.068 |
| Physical Isolation (lower = more isolated) | -0.165 | -0.5 | 0.17 | 0.171 | -0.965 | 14824.8 | 0.334 |
| HADS - Anxiety Score | 0.49 | 0.275 | 0.705 | 0.11 | 4.465 | 5379128.678 | **< .001** |
| Age (years) | -0.052 | -0.097 | -0.007 | 0.023 | -2.257 | 522079.371 | **0.024** |
| Gender (Ref: Male) | 0.008 | -1.548 | 1.564 | 0.794 | 0.01 | 3697415.05 | 0.992 |
| Days in physical isolation | -0.053 | -0.126 | 0.02 | 0.037 | -1.431 | 13079003.177 | 0.152 |
| Distancing Duration (Days Practicing) | -0.035 | -0.075 | 0.006 | 0.021 | -1.659 | 2652345.266 | 0.097 |
| Assessment Point*Social Activity Freq. - Close Contacts | 0.032 | -0.035 | 0.099 | 0.034 | 0.956 | 123.453 | 0.341 |
| Assessment Point*HADS - Depression Score | 0.007 | -0.019 | 0.033 | 0.013 | 0.509 | 207.11 | 0.611 |
| Social Activity Freq. - Close Contacts*HADS - Depression Score | -0.007 | -0.091 | 0.078 | 0.043 | -0.156 | 159.769 | 0.876 |
| Assessment Point*Social Activity Freq. - Close Contacts*HADS - Depression Score | -0.014 | -0.026 | -0.002 | 0.006 | -2.261 | 123.355 | **0.025** |

Note: ^^^ Mental Health Status variables computed relative to reference response level ‘No’.

Table S9a: Effect estimates from imputed models examining the impact of predictor variables on Loneliness scores, for the low depression group (HADS - Depression ≤ 6) only.

| **Predictor** | **Estimate** | **2.5 %** | **97.5 %** | ***SE*** | ***t*** | **df** | ***p*** |
| --- | --- | --- | --- | --- | --- | --- | --- |
| (Intercept) | 16.885 | 12.71 | 21.06 | 2.13 | 7.927 | 31880.304 | **< .001** |
| Assessment Point (Days from Baseline assessment) | -0.033 | -0.144 | 0.077 | 0.056 | -0.596 | 106.525 | 0.553 |
| Social Activity Freq. - Close Contacts | -0.222 | -0.532 | 0.089 | 0.159 | -1.397 | 9940.713 | 0.163 |
| Mental Health Status (‘Not sure’) ^^^ | 7.415 | 2.056 | 12.773 | 2.734 | 2.712 | 50092372.05 | **0.007** |
| Mental Health Status (Current Diagnosis) ^^^ | 1.969 | -0.302 | 4.241 | 1.159 | 1.699 | 6043920.564 | 0.089 |
| Mental Health Status (Past Diagnosis) ^^^ | 2.422 | 0.798 | 4.047 | 0.829 | 2.922 | 29249143.108 | **0.003** |
| Social Activity Freq. - Intermediate Contacts | -0.176 | -0.492 | 0.14 | 0.161 | -1.094 | 615.257 | 0.274 |
| Social Activity Freq. - Distant Contacts | 0.252 | -0.051 | 0.556 | 0.155 | 1.629 | 2258.218 | 0.103 |
| Physical Isolation (lower = more isolated) | -0.324 | -0.681 | 0.032 | 0.182 | -1.782 | 6589.009 | 0.075 |
| HADS - Anxiety Score | 0.741 | 0.516 | 0.966 | 0.115 | 6.457 | 8995038.621 | **< .001** |
| Age (years) | -0.095 | -0.144 | -0.046 | 0.025 | -3.785 | 2595030.671 | **< .001** |
| Gender (Ref: Male) | -0.143 | -1.882 | 1.596 | 0.887 | -0.161 | 1029969.655 | 0.872 |
| Days in physical isolation | -0.021 | -0.098 | 0.055 | 0.039 | -0.55 | 50915271.716 | 0.582 |
| Distancing Duration (Days Practicing) | -0.02 | -0.066 | 0.026 | 0.023 | -0.837 | 236179000.619 | 0.403 |
| Assessment Point*Social Activity Freq. - Close Contacts | 0 | -0.049 | 0.048 | 0.024 | -0.02 | 68.651 | 0.984 |

Note: ^^^ Mental Health Status variables computed relative to reference response level ‘No’.

Table S9b: Effect estimates from imputed models examining the impact of predictor variables on Loneliness scores, for the high depression group (HADS - Depression > 6) only.

| **Predictor** | **Estimate** | **2.5 %** | **97.5 %** | ***SE*** | ***t*** | **df** | ***p*** |
| --- | --- | --- | --- | --- | --- | --- | --- |
| (Intercept) | 18.998 | 8.813 | 29.184 | 5.195 | 3.657 | 3904.407 | **< .001** |
| Assessment Point (Days from Baseline assessment) | 0.053 | -0.201 | 0.308 | 0.13 | 0.411 | 2031.849 | 0.681 |
| Social Activity Freq. - Close Contacts | -0.309 | -1.292 | 0.675 | 0.501 | -0.616 | 524.176 | 0.538 |
| Mental Health Status (Current Diagnosis) ^^^ | 0.239 | -3.909 | 4.388 | 2.115 | 0.113 | 2093.925 | 0.91 |
| Mental Health Status (Past Diagnosis) ^^^ | -2.29 | -6.552 | 1.973 | 2.175 | -1.053 | 139088.424 | 0.292 |
| Social Activity Freq. - Intermediate Contacts | 0.148 | -0.718 | 1.015 | 0.442 | 0.336 | 1226.716 | 0.737 |
| Social Activity Freq. - Distant Contacts | 0.145 | -0.785 | 1.076 | 0.474 | 0.307 | 1390.411 | 0.759 |
| Physical Isolation (lower = more isolated) | 0.09 | -0.798 | 0.979 | 0.453 | 0.2 | 158096.834 | 0.842 |
| HADS - Anxiety Score | 0.915 | 0.406 | 1.424 | 0.26 | 3.522 | 25803.888 | **< .001** |
| Age (years) | 0.096 | -0.027 | 0.219 | 0.063 | 1.53 | 10583.186 | 0.126 |
| Gender (Ref: Male) | -1.003 | -5.052 | 3.045 | 2.065 | -0.486 | 94033.899 | 0.627 |
| Days in physical isolation | -0.325 | -0.575 | -0.075 | 0.128 | -2.544 | 163964.491 | **0.011** |
| Distancing Duration (Days Practicing) | -0.086 | -0.187 | 0.015 | 0.052 | -1.675 | 15919.683 | 0.094 |
| Assessment Point*Social Activity Freq. - Close Contacts | -0.129 | -0.252 | -0.005 | 0.063 | -2.05 | 289.656 | **0.041** |

Note: ^^^ Mental Health Status variables computed relative to reference response level ‘No’.

Table S10: Effect estimates from imputed models examining the impact of predictor variables on Loneliness scores.

| **Predictor** | **Estimate** | **2.5 %** | **97.5 %** | ***SE*** | ***t*** | **df** | ***p*** |
| --- | --- | --- | --- | --- | --- | --- | --- |
| (Intercept) | 12.686 | 8.852 | 16.52 | 1.956 | 6.486 | 42514.793 | **< .001** |
| Assessment Point (Days from Baseline assessment) | -0.028 | -0.161 | 0.104 | 0.068 | -0.42 | 463.606 | 0.675 |
| Social Activity Freq. - Intermediate Contacts | -0.093 | -0.55 | 0.365 | 0.233 | -0.397 | 1239.759 | 0.692 |
| HADS - Depression Score | 1.312 | 1.021 | 1.604 | 0.149 | 8.829 | 2498.297 | **< .001** |
| Mental Health Status (‘Not sure’) ^^^ | 8.799 | 3.313 | 14.285 | 2.799 | 3.144 | 281657222.34 | **0.002** |
| Mental Health Status (Current Diagnosis) ^^^ | 2.164 | 0.281 | 4.047 | 0.961 | 2.253 | 937784.794 | **0.024** |
| Mental Health Status (Past Diagnosis) ^^^ | 1.913 | 0.413 | 3.414 | 0.766 | 2.499 | 9896307.995 | **0.012** |
| Social Activity Freq. - Close Contacts | -0.387 | -0.69 | -0.084 | 0.154 | -2.505 | 663.613 | **0.012** |
| Social Activity Freq. - Distant Contacts | 0.28 | -0.021 | 0.58 | 0.153 | 1.824 | 5035.346 | 0.068 |
| Physical Isolation (lower = more isolated) | -0.161 | -0.497 | 0.175 | 0.172 | -0.939 | 11487.397 | 0.348 |
| HADS - Anxiety Score | 0.49 | 0.275 | 0.705 | 0.11 | 4.459 | 7810294.708 | **< .001** |
| Age (years) | -0.051 | -0.095 | -0.006 | 0.023 | -2.21 | 1372284.457 | **0.027** |
| Gender (Ref: Male) | 0.053 | -1.502 | 1.608 | 0.793 | 0.067 | 8664709.248 | 0.947 |
| Days in physical isolation | -0.054 | -0.126 | 0.019 | 0.037 | -1.443 | 16283236.085 | 0.149 |
| Distancing Duration (Days Practicing) | -0.034 | -0.075 | 0.007 | 0.021 | -1.624 | 3489267.424 | 0.104 |
| Assessment Point*Social Activity Freq. - Intermediate Contacts | 0.016 | -0.044 | 0.076 | 0.031 | 0.517 | 340.736 | 0.606 |
| Assessment Point*HADS - Depression Score | -0.003 | -0.027 | 0.02 | 0.012 | -0.255 | 191.313 | 0.799 |
| Social Activity Freq. - Intermediate Contacts*HADS - Depression Score | 0.01 | -0.063 | 0.084 | 0.038 | 0.279 | 796.61 | 0.78 |
| Assessment Point*Social Activity Freq. - Intermediate Contacts*HADS - Depression Score | -0.008 | -0.02 | 0.004 | 0.006 | -1.249 | 169.37 | 0.214 |

Note: ^^^ Mental Health Status variables computed relative to reference response level ‘No’.

Table S11: Effect estimates from imputed models examining the impact of predictor variables on Loneliness scores.

| **Predictor** | **Estimate** | **2.5 %** | **97.5 %** | ***SE*** | ***t*** | **df** | ***p*** |
| --- | --- | --- | --- | --- | --- | --- | --- |
| (Intercept) | 12.867 | 9.037 | 16.698 | 1.954 | 6.584 | 26311.21 | **< .001** |
| Assessment Point (Days from Baseline assessment) | -0.057 | -0.177 | 0.063 | 0.061 | -0.936 | 205.569 | 0.35 |
| Social Activity Freq. - Distant Contacts | 0.176 | -0.304 | 0.657 | 0.245 | 0.72 | 4408.351 | 0.472 |
| HADS - Depression Score | 1.298 | 1.042 | 1.554 | 0.131 | 9.928 | 15914.555 | **< .001** |
| Mental Health Status (‘Not sure’) ^^^ | 8.788 | 3.302 | 14.275 | 2.799 | 3.139 | 118481869.287 | **0.002** |
| Mental Health Status (Current Diagnosis) ^^^ | 2.173 | 0.291 | 4.055 | 0.96 | 2.263 | 726508.019 | **0.024** |
| Mental Health Status (Past Diagnosis) ^^^ | 1.92 | 0.42 | 3.42 | 0.765 | 2.508 | 11385296.894 | **0.012** |
| Social Activity Freq. - Close Contacts | -0.39 | -0.692 | -0.089 | 0.154 | -2.541 | 717.555 | **0.011** |
| Social Activity Freq. - Intermediate Contacts | -0.096 | -0.407 | 0.215 | 0.158 | -0.604 | 552.996 | 0.546 |
| Physical Isolation (lower = more isolated) | -0.16 | -0.495 | 0.176 | 0.171 | -0.933 | 11488.048 | 0.351 |
| HADS - Anxiety Score | 0.49 | 0.275 | 0.706 | 0.11 | 4.462 | 4447829.915 | **< .001** |
| Age (years) | -0.05 | -0.095 | -0.005 | 0.023 | -2.187 | 3704509.796 | **0.029** |
| Gender (Ref: Male) | 0.035 | -1.521 | 1.591 | 0.794 | 0.044 | 2653359.929 | 0.965 |
| Days in physical isolation | -0.054 | -0.126 | 0.019 | 0.037 | -1.45 | 16847638.168 | 0.147 |
| Distancing Duration (Days Practicing) | -0.035 | -0.076 | 0.006 | 0.021 | -1.659 | 11347061.088 | 0.097 |
| Assessment Point*Social Activity Freq. - Distant Contacts | 0.043 | -0.023 | 0.108 | 0.034 | 1.268 | 656.165 | 0.205 |
| Assessment Point*HADS - Depression Score | -0.001 | -0.022 | 0.02 | 0.01 | -0.116 | 81.246 | 0.908 |
| Social Activity Freq. - Distant Contacts*HADS - Depression Score | 0.03 | -0.06 | 0.119 | 0.046 | 0.652 | 2898.116 | 0.514 |
| Assessment Point*Social Activity Freq. - Distant Contacts*HADS - Depression Score | -0.013 | -0.026 | 0.001 | 0.007 | -1.808 | 275.352 | 0.072 |

Note: ^^^ Mental Health Status variables computed relative to reference response level ‘No’.
